# Microarray analysis of microRNA expression in mouse fetus at 13.5 and 14.5 days *post*-*coitum* in ear and back skin tissues

**DOI:** 10.1016/j.gdata.2016.06.011

**Published:** 2016-06-24

**Authors:** Leda Torres, Ulises Juárez, Laura García, Juan Miranda-Ríos, Sara Frias

**Affiliations:** a Laboratorio de Citogenética, Depto. de Investigación en Genética Humana, Instituto Nacional de Pediatría, Ciudad de México, México; b Posgrado en Ciencias Biológicas, Universidad Nacional Autónoma de México, Ciudad de México, México; c Posgrado en Ciencias Biomédicas, Universidad Nacional Autónoma de México, Ciudad de México, México; d Unidad de Genética de la Nutrición, Instituto de Investigaciones Biomédicas, Universidad Nacional Autónoma de México e Instituto Nacional de Pediatría, Ciudad de México, México

**Keywords:** Microarray, miRNAs, Mouse, Ear development

## Abstract

There is no information regarding the role of microRNAs in the development of the external ear in mammals. The purpose of this study was to determine the stage-specific expression of microRNA during external ear development in mice under normal conditions. GeneChip miRNA 3.0 arrays by Affymetrix were used to obtain miRNA expression profiles from mice fetal pinnae and back skin tissues at 13.5 days-*post*-*coitum* (dpc) and 14.5 dpc. Biological triplicates for each tissue were analyzed; one litter represents one biological replica, each litter had 16 fetuses on average. The results were analyzed with Affymetrix's Transcriptome Analysis Console software to identify differentially expressed miRNAs. The inquiry showed significant differential expression of 25 miRNAs at 13.5 dpc and 31 at 14.5 dpc, some of these miRNAs were predicted to target genes implicated in external ear development. One example is mmu-miR-10a whose low expression in pinnae is known to impact ear development by modulating *Hoxa1* mRNA levels Garzon et al. (2006), Gavalas et al. (1998) [1], [2]. Other findings like the upregulation of mmu-miR-200c and mmu-miR-205 in the pinnae tissues of healthy mice are in agreement with what has been reported in human patients with microtia, in which down regulation of both miRNAs has been found Li et al. (2013) [3].

This study uncovered a spatiotemporal pattern of miRNA expression in the external ear, which results from continuous transcriptional changes during normal development of body structures.

All microarray data are available at the Gene Expression Omnibus (GEO) at NCBI under accession number GSE64945.

**Table:** 

Specifications	

Organism/cell line/tissue	*Mus musculus* CD1 strain/fetuses/pinnae and back skin
Sex	Male and female
Sequencer or array type	Affymetrix GeneChip miRNA 3.0 array
Data format	Raw CEL and CHP files
Experimental factors	Developmental stages: 13.5 and 14.5 dpc
Experimental features	Total RNA from external ear tissue, the pinnae (cartilage and skin), and back skin tissues of each developmental stage was extracted and subjected to microarray analysis. The raw data were analyzed with Expression Console and Transcriptome Analysis Console by Affymetrix.
Consent	Approved by the Institutional committee of Care and Use of Laboratory Animals, Instituto Nacional de Pediatría, México.
Sample source location	Not applicable

## 1 Direct link to deposited data

The data are available at GEO public repository:

*http:*//*www.ncbi.nlm.nih.gov*/*geo*/*query*/*acc.cgi?acc*=*GSE64945*

## 2 Experimental design, materials and methods

### 2.1 Mice

In this study we worked with CD1 mice strain. The mice habitat conditions were controlled (12 h:12 h light/dark cycle, temperature 19.5–22.5 °C, relative humidity 45–55%) and the mice were provided with water and food (Teklab Global Rodent Diet, Harlan) ad libitum. All management and experimental procedures were in accordance with the Mexican regulation norm NOM-062-ZOO-1999 and the Guide for Care and Use of Laboratory Animals, Institute for Laboratory Animal Research of the National Academy of Sciences, USA. The Institutional Animal Care and Use of Laboratory Animals and Research Committees from the Instituto Nacional de Pediatría, approved this study.

Scheduled mating was carried out in order to obtain fetuses at specific developmental stages. Pregnant females were sacrificed by cervical dislocation at 13.5 dpc or 14.5 dpc. The gestational age of the fetus was determined by the day of appearance of the vaginal plug (0.5 dpc) and confirmed by the following morphological criteria:

**Table:** 

Gestational age	Somite pair count	Limbs	Fur
13.5	52–55	Distal separation of the fingers	Absence of hair follicles
14.5	56–60	Proximal separation of the fingers	Sparse facial hair

### 2.2 Tissues

Biological triplicates for each tissue and each developmental stage were analyzed. One litter represents one biological replica and each litter had an average of 16 fetuses (32 pinnae). External ear tissue, the pinnae (cartilage and skin), and back skin tissues were dissected under stereomicroscope in iced cooled nuclease free PBS (Gibco, Waltham, MA USA) and were kept in RNAlater (Ambion, Waltham, MA USA) at -80 °C until further processing. All tissues belonging to a specific body region from a particular developmental stage were pooled in a single sample per biological replica; this procedure was very successful to obtain enough tissue from this anatomically small region.

### 2.3 microRNA array analysis

Total RNA including short RNAs (< 200 bp) was extracted with an RNA Mini elute kit (Qiagen, Venlo, Netherlands) and quantitated with a NanodropTM spectrophotometer (Nanodrop Technologies, Wilmington, DE, USA). RNA quality was monitored by agarose gel electrophoresis.

miRNA profiling in the extracted RNA was performed by using the Affymetrix GeneChip miRNA Arrays 3.0 (Affymetrix, Santa Clara, CA, USA). In this version of the array the manufacturer includes 179,217 probes that represent 19,913 mature miRNA contained in miRBase V.17 (www.mirbase.org) with probes for 153 different organisms, including 1111 probes for *Mus musculus* miRNAs and 855 pre-miRNAs. We performed the arrays according to Affymetrix instructions. Briefly, 500 ng of total RNA, were labeled with the FlashTag ® Biotin HSR kit (Genisphere/Affymetrix, Santa Clara, CA, USA). Correct labeling was confirmed by Enzyme Linked Oligosorbent Assay (ELOSA) QC Assay (Thermo Fisher Scientific Inc., Waltham, MA, USA). Samples were denaturalized at 99 °C for 5 min followed by 45 °C for another 5 min, injected into the array chips and allowed hybridization for 17 h at 48 °C in an Affymetrix Hybridization Oven 645 in constant movement at 60 rpm. Arrays were then stained and washed in the Affymetrix GeneChip Fluidic Station 450 and scanned using an Affymetrix GeneChip Scanner 3000 7G (Affymetrix, Santa Clara, CA, USA).

**Fig. 1:**
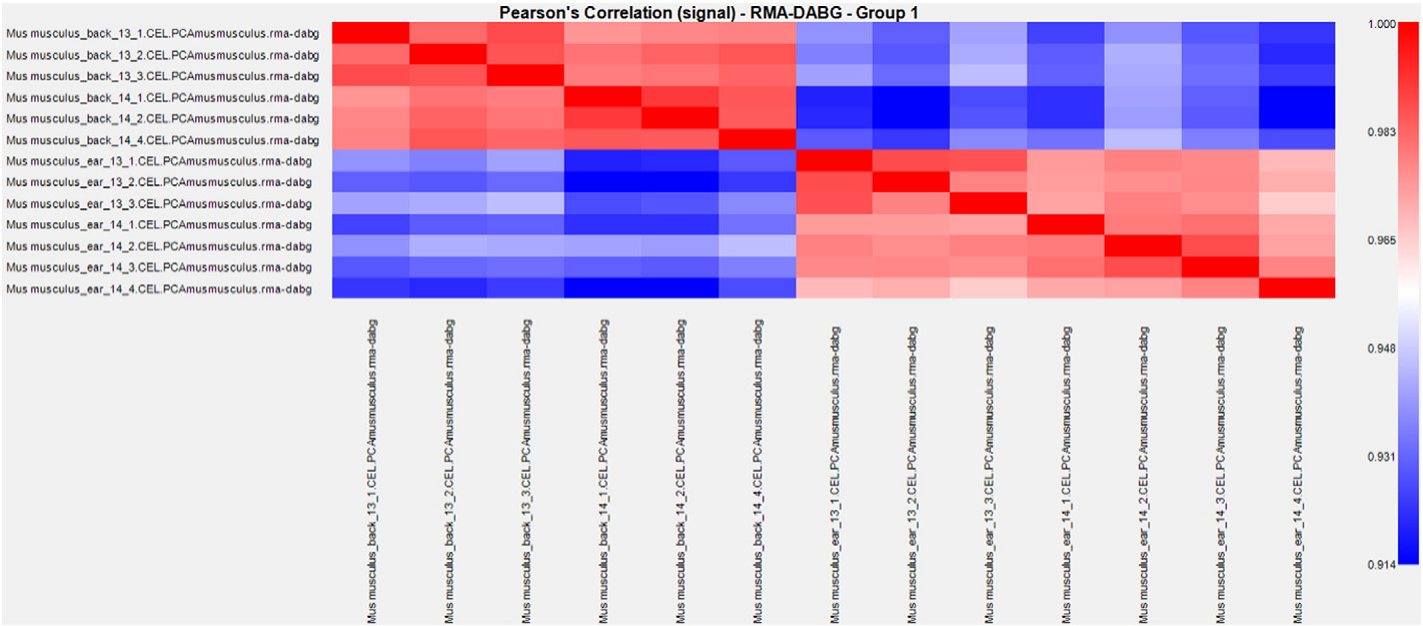
Pearson's Correlation of the signal obtained from Affymetrix miRNA Arrays 3.0 GeneChips hybridized.

**Fig. 2:**
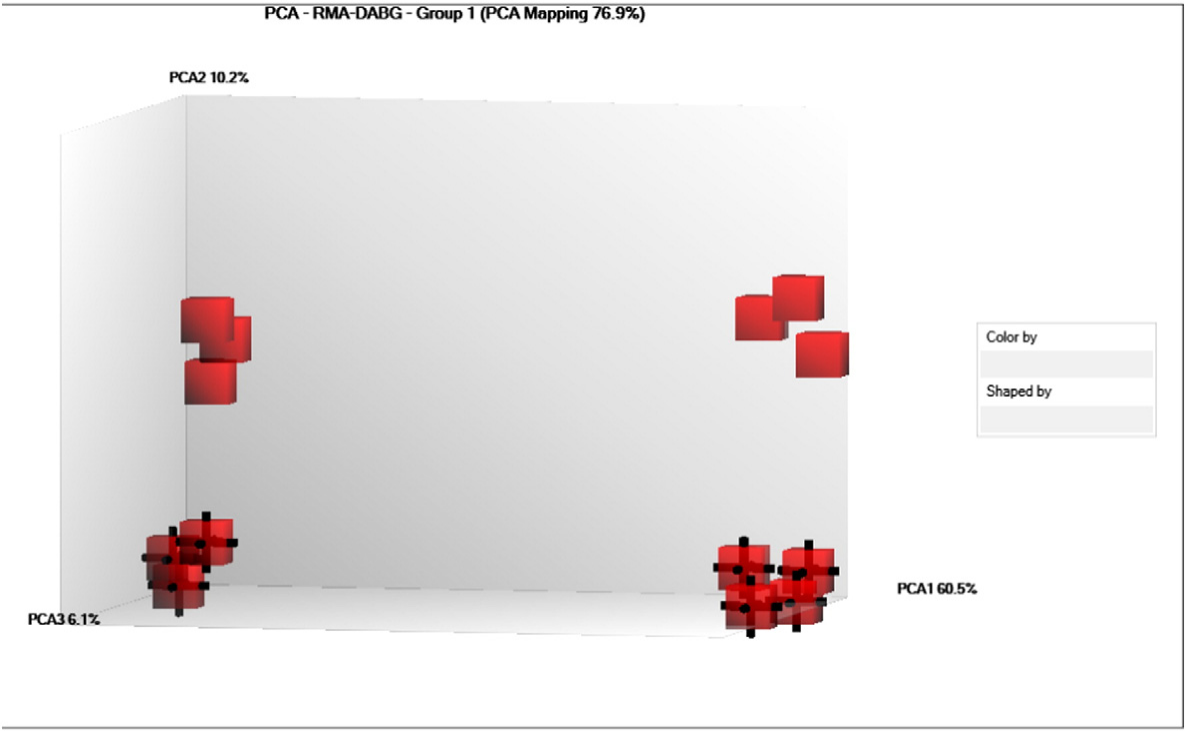
Loading plot showing the components from Principal Component Analysis (PCA) of all probe sets from Affymetrix GeneChip miRNA Arrays 3.0 hybridized. We clearly observed four groups that can be arranged by developmental stage (red cubes 13.5 dpc, red cubes with black edges 14.5 dpc) or by tissue (right side pinnae, left side back skin).

**Fig. 3:**
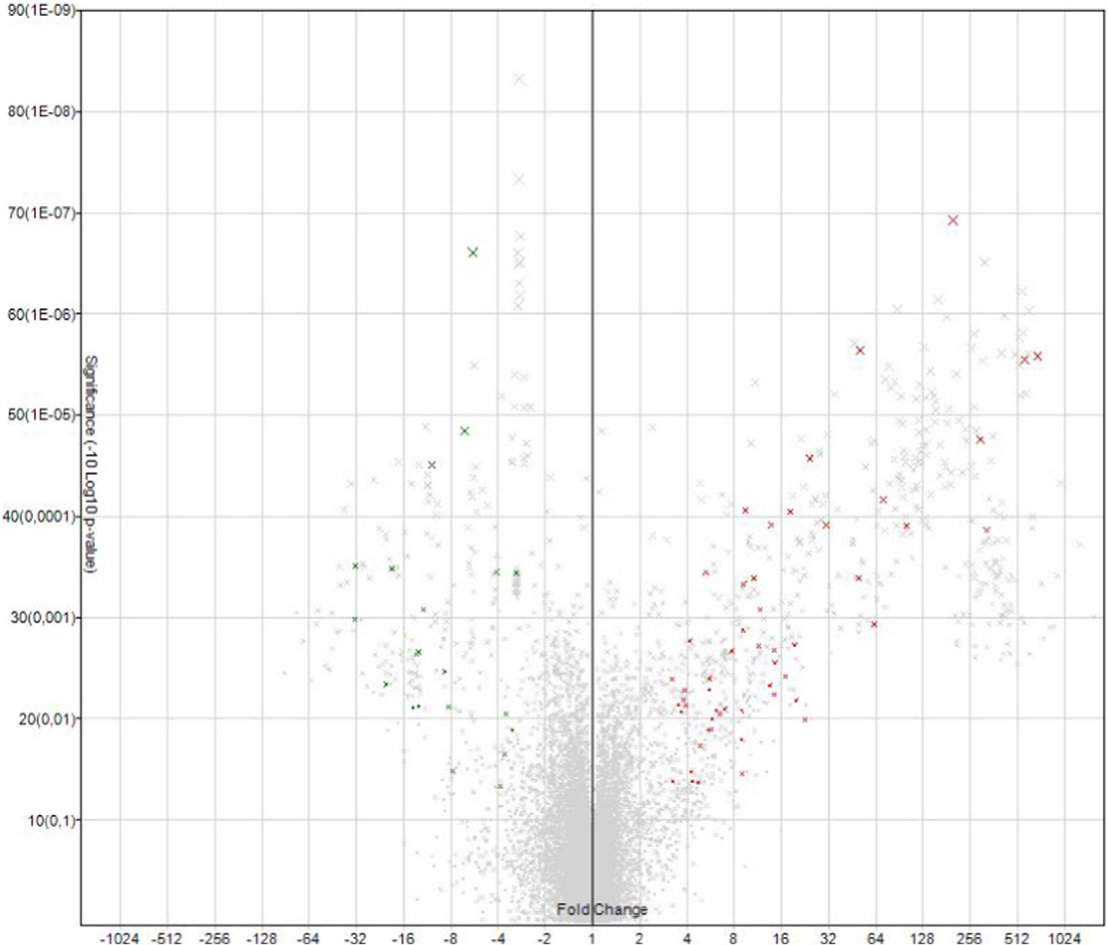
Volcano plot from GeneChip miRNA Arrays 3.0 analysis at 13.5 dpc, expression levels comparing ear (pinnae) and back tissues. Fold change: + 3 and − 3, Adjusted P-value threshold 0.05. Significant spots in green and red, green X: low expression levels in back skin, red X: high expression levels in pinnae.

**Fig. 4:**
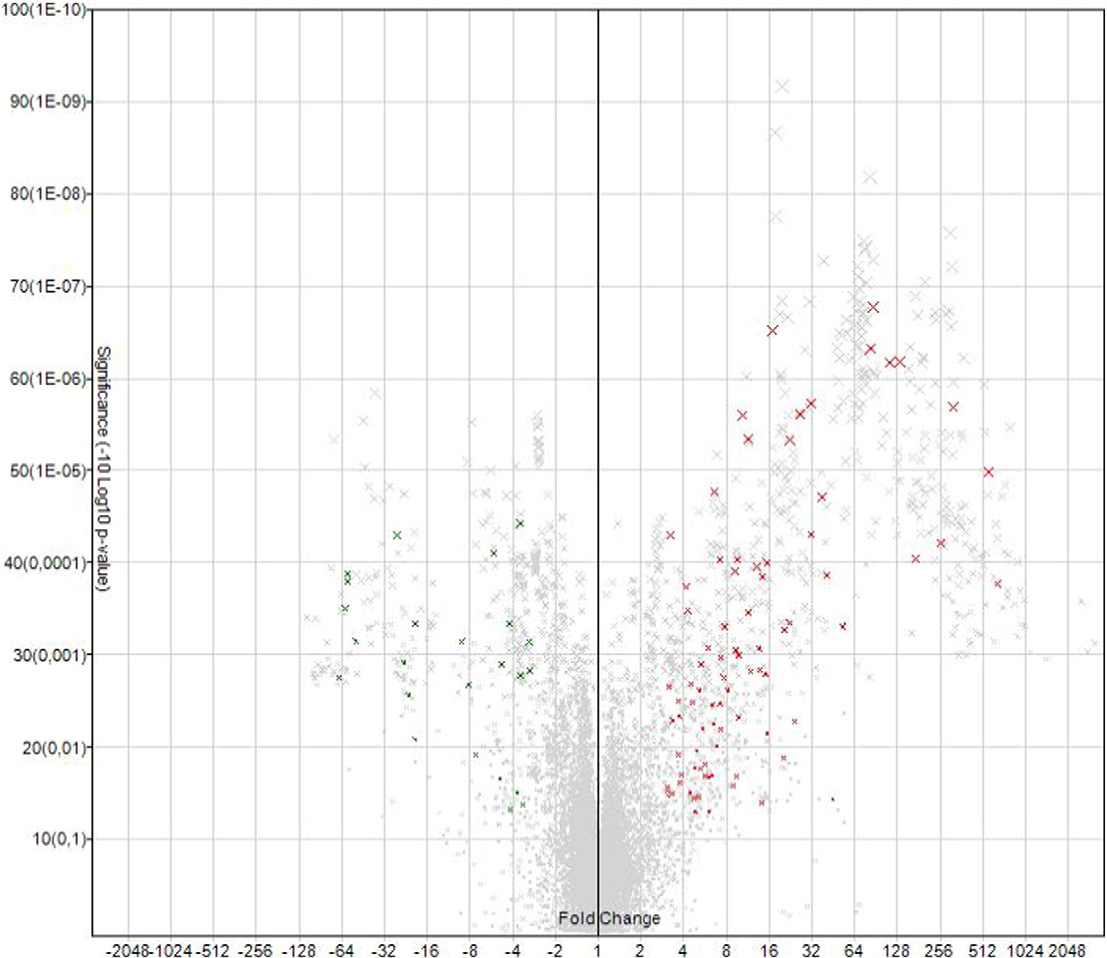
Volcano plot from GeneChip miRNA Arrays 3.0 analysis at 14.5 dpc, expression levels comparing ear (pinnae) and back tissues. Fold change: + 3 and − 3, Adjusted P-value threshold 0.05. Significant spots in green and red, green X: low expression levels in back skin, red X: high expression levels in pinnae.

**Fig. 5:**
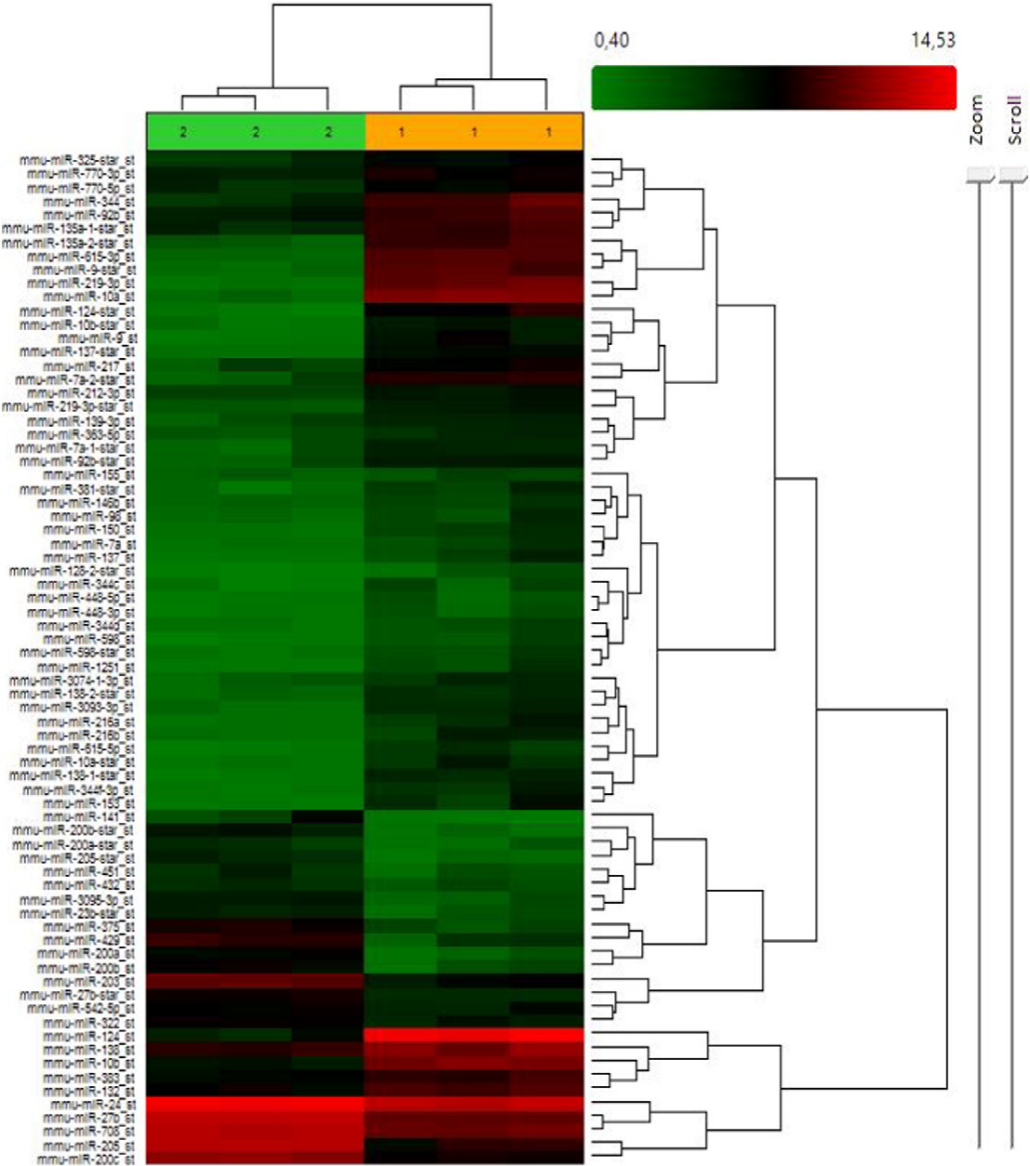
Heat map from GeneChip miRNA Arrays 3.0 analysis at 13.5 dpc, expression levels comparing ear (pinnae)(2) and back tissues(1). Fold change: + 3 and − 3, Adjusted P-value threshold 0.05. Green low expression levels in back skin, red high expression levels in pinnae.

**Fig. 6:**
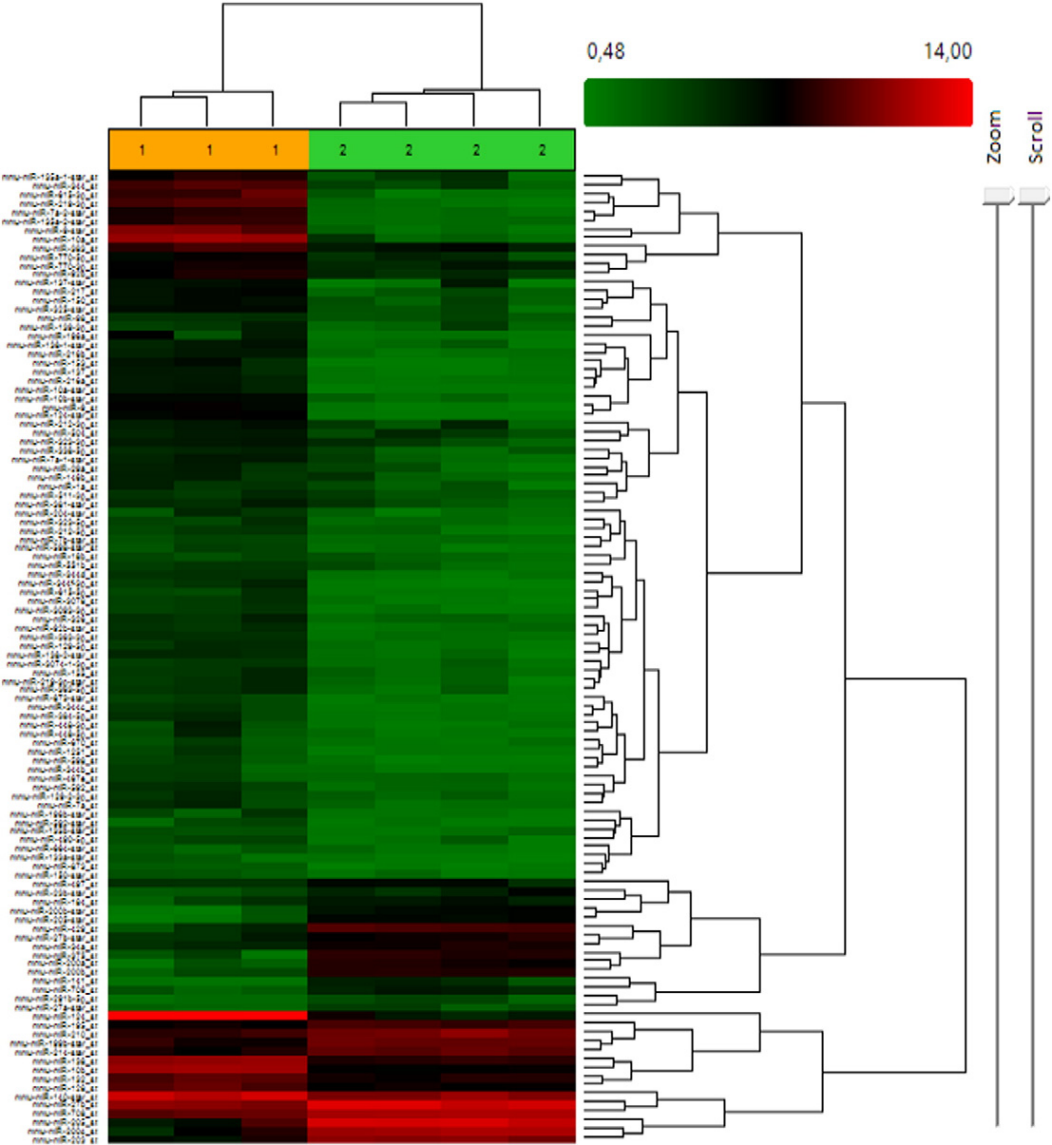
Heat map from GeneChip miRNA Arrays 3.0 analysis at 14.5 dpc, expression levels comparing ear (pinnae)(2) and back tissues (1). Fold change: + 3 and − 3, Adjusted P-value threshold 0.05. Green low expression levels in back skin, red high expression levels in pinnae.

The miRNA QC tool was used to assess the quality of the array data, afterwards data were normalized by global normalization with Robust Multichip Average (RMA) and Detection Above Background (DABG) using of the Expression Console software (Affymetrix, Santa Clara, CA, USA) (Fig. 1, Fig. 2). The identification of differentially expressed miRNAs at each stage, was achieved by using linear regression and hierarchical clustering analysis with the Transcriptome Analysis Console software from Affymetrix. The filter settings were the following: “Transcription Cluster ID”: contains mmu-miR does not contain hp.-mmu-miR, “Fold change”: + 3 and − 3, “False discovered rate (FDR)”: 1.0. “Adjusted P-value threshold”: 0.05. Expression levels of the miRNAs were compared between ear and back tissues (Fig. 3, Fig. 4, Fig. 5, Fig. 6) (Table 1, Table 2).

Putative mRNA targets for differentially expressed miRNAs were identified using the bioinformatic predicting tools TargetScanMouse Release 6.2 (www.targetscan.org) miRNA body Map (www.mirnabodymap.org), miRBase (www.mirbase.org) and DIANA Tools (Tarbase, microT v4 and microT-CDS), (http://diana.imis.athena-innovation.gr/DianaTools) (Table 3).

## 3 Discussion

This data set results from the first effort to obtain miRNA expression profiling data of external ear development in mammals. This data should prove useful to understand the role of miRNAs in normal external ear development in mammals and might hint over miRNA involvement in the etiology of microtia [4] Data was validated by Whole mount in situ hybridization in a subset of miRNAs whose mRNA targets have been associated with external ear development and present clear differential spatiotemporal expression patterns (mmu-miR-10a, mmu-miR-200c and mmu-miR-205) [1], [2], [3], [5]. The microarray data presented in this work has been deposited in Gene Expression Omnibus through GEO Series with accession number GSE64945.

## Conflict of interest

The authors declare no conflict of interest.

## Figures and Tables

**Table 1:** Differentially expressed microRNA in ear (pinnae) versus back skin at 13.5 dpc.

microRNA ID	Ear 13.5 dpc	Back 13.5 dpc	Fold change
mmu-miR-200a_st	7.14	2.1	32.85
mmu-miR-200b-star_st	6.23	1.21	32.44
mmu-miR-200b_st	7.41	3.03	20.76
mmu-miR-375_st	8.09	3.84	19
mmu-miR-200a-star_st	5.13	1.32	14.01
mmu-miR-3095-3p_st	6.07	2.35	13.25
mmu-miR-429_st	8.54	4.85	12.96
mmu-miR-205_st	12.44	8.75	12.85
mmu-miR-205-star_st	5.31	1.72	12.05
mmu-miR-200c_st	11.28	7.86	10.67
mmu-miR-203_st	10.07	6.94	8.75
mmu-miR-23b-star_st	5.82	2.77	8.3
mmu-miR-141_st	3.93	0.97	7.79
mmu-miR-27b-star_st	7.92	5.22	6.53
mmu-miR-27b_st	12.69	10.15	5.82
mmu-miR-708_st	12.39	10.36	4.1
mmu-miR-542-5p_st	7.45	5.5	3.88
mmu-miR-451_st	4.68	2.82	3.63
mmu-miR-432_st	4.98	3.14	3.56
mmu-miR-322_st	7.65	5.95	3.25
mmu-miR-24_st	14.13	12.52	3.05
mmu-miR-383_st	7.14	8.82	− 3.2
mmu-miR-155_st	2.1	3.8	− 3.26
mmu-miR-344d_st	1.39	3.21	− 3.53
mmu-miR-598_st	0.8	2.67	− 3.65
mmu-miR-598-star_st	1.29	3.21	− 3.78
mmu-miR-132_st	7.48	9.44	− 3.89
mmu-miR-146b_st	1.91	3.9	− 3.95
mmu-miR-770-3p_st	5.78	7.85	− 4.2
mmu-miR-448-5p_st	0.86	2.95	− 4.25
mmu-miR-98_st	1.58	3.7	− 4.34
mmu-miR-128-2-star_st	0.57	2.81	− 4.73
mmu-miR-448-3p_st	0.89	3.16	− 4.83
mmu-miR-212-3p_st	3.91	6.3	− 5.27
mmu-miR-139-3p_st	3.12	5.57	− 5.47
mmu-miR-363-5p_st	2.87	5.34	− 5.54
mmu-miR-138_st	8.61	11.1	− 5.58
mmu-miR-7a_st	1.26	3.78	− 5.72
mmu-miR-381-star_st	1.76	4.29	− 5.79
mmu-miR-770-5p_st	4.71	7.33	− 6.15
mmu-miR-1251_st	0.86	3.56	− 6.47
mmu-miR-325-star_st	4.31	7.11	− 6.99
mmu-let-7b_st	11.04	13.96	− 7.6
mmu-miR-150_st	1.33	4.28	− 7.71
mmu-miR-137_st	1.07	4.22	− 8.88
mmu-miR-3074-1-3p_st	2.34	5.5	− 8.91
mmu-miR-344c_st	0.72	3.88	− 8.95
mmu-miR-92b_st	5.93	9.12	− 9.09
mmu-miR-138-2-star_st	1.93	5.13	− 9.19
mmu-miR-219-3p-star_st	2.78	6.02	− 9.41
mmu-miR-615-5p_st	0.81	4.22	− 10.65
mmu-miR-344f-3p_st	1.03	4.55	− 11.47
mmu-miR-135a-1-star_st	5.4	8.95	− 11.73
mmu-miR-216a_st	1.32	5.07	− 13.5
mmu-miR-3093-3p_st	1.43	5.2	− 13.68
mmu-miR-10a-star_st	1.12	4.97	− 14.42
mmu-miR-7a-1-star_st	1.67	5.53	− 14.47
mmu-miR-217_st	3.47	7.33	− 14.51
mmu-miR-92b-star_st	1.99	6.08	− 17.04
mmu-miR-10b_st	6.63	10.81	− 18.21
mmu-miR-344_st	4.8	9.08	− 19.38
mmu-miR-153_st	0.86	5.18	− 19.92
mmu-miR-216b_st	1.42	5.91	− 22.6
mmu-miR-138-1-star_st	0.88	5.47	− 24.21
mmu-miR-10b-star_st	0.93	5.88	− 30.85
mmu-miR-9_st	0.87	6.51	− 49.6
mmu-miR-137-star_st	1.27	6.93	− 50.77
mmu-miR-7a-2-star_st	2.48	8.45	− 62.68
mmu-miR-135a-2-star_st	2.68	8.84	− 71.33
mmu-miR-124-star_st	0.81	7.46	− 99.99
mmu-miR-615-3p_st	1.96	9.58	− 197.55
mmu-miR-9-star_st	1.51	9.72	− 296.06
mmu-miR-124_st	5.85	14.2	− 325.39
mmu-miR-219-3p_st	1.12	10.27	− 565.86
mmu-miR-10a_st	1.57	10.99	− 684.75

**Table 2:** Differentially expressed microRNA in ear (pinnae) versus back skin at 14.5 dpc.

microRNA ID	Ear 14.5 dpc	Back 14.5 dpc	Fold change
mmu-miR-205_st	12.87	6.8	67.04
mmu-miR-200b-star_st	6.78	0.86	60.56
mmu-miR-205-star_st	6.94	1.08	58.14
mmu-miR-200a_st	7.93	2.07	57.91
mmu-miR-375_st	8.27	2.6	50.82
mmu-miR-200b_st	8.2	3.49	26.15
mmu-miR-429_st	9.1	4.54	23.51
mmu-miR-200c_st	11.6	7.16	21.63
mmu-miR-141_st	5.43	1.14	19.67
mmu-miR-203_st	10.84	6.56	19.51
mmu-miR-706_st	5.61	2.41	9.15
mmu-miR-27b-star_st	7.65	4.63	8.14
mmu-miR-23b-star_st	5.39	2.52	7.31
mmu-miR-34a_st	7.75	5.3	5.48
mmu-miR-184_st	5.98	3.69	4.89
mmu-miR-199b-star_st	9.91	7.65	4.79
mmu-miR-27b_st	12.88	10.81	4.19
mmu-miR-497_st	6.72	4.66	4.17
mmu-miR-291b-5p_st	3.56	1.67	3.72
mmu-miR-708_st	11.69	9.86	3.56
mmu-miR-210_st	10.18	8.37	3.52
mmu-miR-27a-star_st	3.66	1.9	3.4
mmu-miR-195_st	9.07	7.45	3.07
mmu-miR-214-star_st	9.69	8.08	3.05
mmu-miR-18b_st	2.03	3.64	− 3.04
mmu-miR-664-star_st	1.04	2.66	− 3.06
mmu-miR-132_st	7.92	9.58	− 3.15
mmu-miR-133a-star_st	0.82	2.47	− 3.15
mmu-miR-873_st	0.71	2.39	− 3.22
mmu-let-7b_st	12.01	13.74	− 3.3
mmu-miR-150-star_st	1.03	2.76	− 3.32
mmu-miR-511-3p_st	2.84	4.58	− 3.33
mmu-miR-670_st	1.21	3.07	− 3.63
mmu-miR-135b-star_st	1.18	3.06	− 3.68
mmu-miR-98_st	2.78	4.68	− 3.73
mmu-miR-1a_st	2.63	4.54	− 3.78
mmu-miR-448-3p_st	0.93	2.88	− 3.84
mmu-miR-7b-star_st	1.6	3.67	− 4.2
mmu-miR-128_st	7.37	9.47	− 4.28
mmu-miR-504_st	3.82	5.97	− 4.43
mmu-miR-323-5p_st	1.67	3.84	− 4.5
mmu-miR-140-star_st	10.51	12.73	− 4.64
mmu-miR-381-star_st	2.87	5.12	− 4.75
mmu-miR-196b-star_st	1.22	3.49	− 4.8
mmu-miR-467e_st	1.89	4.16	− 4.8
mmu-miR-139-3p_st	1.78	4.07	− 4.9
mmu-miR-129-2-3p_st	2.04	4.36	− 4.97
mmu-miR-323-3p_st	3.61	5.96	− 5.11
mmu-miR-770-3p_st	5.39	7.76	− 5.17
mmu-miR-551b_st	2.02	4.42	− 5.25
mmu-miR-155_st	1.86	4.27	− 5.29
mmu-miR-1251_st	1.07	3.51	− 5.42
mmu-miR-448-5p_st	0.92	3.42	− 5.66
mmu-miR-770-5p_st	4.97	7.48	− 5.71
mmu-miR-615-5p_st	0.9	3.48	− 5.94
mmu-miR-204-star_st	1.52	4.1	− 6.02
mmu-miR-212-3p_st	3.46	6.06	− 6.06
mmu-miR-592-star_st	0.84	3.51	− 6.35
mmu-miR-598-star_st	1.21	3.88	− 6.36
mmu-miR-212-5p_st	1.52	4.22	− 6.5
mmu-miR-138_st	8.31	11.04	− 6.63
mmu-miR-490-5p_st	1.01	3.78	− 6.85
mmu-miR-873-star_st	0.94	3.79	− 7.21
mmu-miR-598_st	0.75	3.61	− 7.22
mmu-miR-3074-1-3p_st	1.41	4.27	− 7.27
mmu-miR-338-5p_st	2.41	5.28	− 7.33
mmu-miR-383_st	6.16	9.09	− 7.65
mmu-miR-326_st	1.87	4.83	− 7.8
mmu-miR-219-3p-star_st	1.62	4.65	− 8.18
mmu-miR-344b_st	0.92	4.09	− 8.99
mmu-miR-92b-star_st	1.45	4.64	− 9.16
mmu-miR-3078_st	0.85	4.05	− 9.18
mmu-miR-92b_st	4.88	8.09	− 9.28
mmu-miR-29a_st	2.22	5.45	− 9.37
mmu-miR-3093-3p_st	0.83	4.08	− 9.5
mmu-miR-7a_st	1.53	4.81	− 9.67
mmu-miR-592_st	1.59	4.88	− 9.77
mmu-miR-363-3p_st	1.37	4.74	− 10.34
mmu-miR-344d_st	1.16	4.68	− 11.45
mmu-miR-344c_st	0.88	4.4	− 11.5
mmu-miR-363-5p_st	1.18	4.74	− 11.81
mmu-miR-129-5p_st	1.31	5.04	− 13.21
mmu-miR-384-5p_st	1.11	4.86	− 13.49
mmu-miR-150_st	2.64	6.44	− 13.86
mmu-miR-146b_st	1.87	5.69	− 14.16
mmu-miR-138-2-star_st	1.3	5.14	− 14.35
mmu-miR-325-star_st	2.86	6.77	− 15.09
mmu-miR-7a-1-star_st	1.9	5.84	− 15.38
mmu-miR-344f-3p_st	0.65	4.61	− 15.65
mmu-miR-10b_st	7.3	11.38	− 16.94
mmu-miR-196a_st	1.16	5.51	− 20.32
mmu-miR-138-1-star_st	1.49	5.85	− 20.52
mmu-miR-217_st	2.42	6.9	− 22.28
mmu-miR-216b_st	1.48	5.96	− 22.35
mmu-miR-135a-1-star_st	3.47	8.08	− 24.38
mmu-miR-216a_st	1.24	5.96	− 26.29
mmu-miR-137_st	0.99	5.97	− 31.56
mmu-miR-10a-star_st	0.87	5.86	− 31.75
mmu-miR-10b-star_st	1.68	6.91	− 37.5
mmu-miR-153_st	0.69	6.03	− 40.5
mmu-miR-137-star_st	0.84	6.33	− 44.98
mmu-miR-344_st	3.49	9.2	− 52.37
mmu-miR-9_st	0.79	7.16	− 82.76
mmu-miR-124-star_st	0.92	7.36	− 86.83
mmu-miR-7a-2-star_st	1.46	8.27	− 112.48
mmu-miR-135a-2-star_st	1.56	8.61	− 132.71
mmu-miR-615-3p_st	1.13	8.55	− 171.31
mmu-miR-124_st	5.98	13.98	− 256.55
mmu-miR-219-3p_st	1.27	9.57	− 316.41
mmu-miR-9-star_st	1.4	10.54	− 562.1
mmu-miR-10a_st	1.83	11.18	− 651.42

**Table 3:** Putative mRNA targets for differentially expressed miRNAs.

	Bioinformatic predicting tools/mmu-miR differentially expressed			
Target Gene	miRNA body Map v1.1	microT v4	microT-CDS v5	Tarbase
*Bmp5*	mmu-miR-203	–	–	–
*Cyp26b1*	mmu-miR-24, 200a, 205, 9, 217	–	mmu-miR-195, 200ª	–
*Dlx5*	mmu-miR-124, 203, 200a	–	mmu-miR-124	mmu-miR-200a
*Dlx6*	mmu-miR-205	–	mmu-miR-7a-2-star	–
*Edn1*	mmu-miR-203	–	mm-miR-344d, 135a-2-star	–
*Ednra*	mmu-miR-203, 27b, 324-3p, 497, 195, 124	–	mmu-miR-27b	mmu-miR-324-3p
*Eya1*	mmu-miR-23b, 27b, 200a, 203, 205, 497, 195, 124, 217	mmu-miR-27b	mmu-miR-124, 10a-star, 195, 497, 27b	–
*Fgf8*	mmu-miR-195	–	–	–
*Fgf10*	mmu-miR-9, 200c, 200b, 203, 375, 429, 137, 217, 344	–	mmu-miR-137, 124, 9	–
*Frem2*	mmu-miR-9, 200b, 24, 205, 27b, 200c, 429, 216a,	–	mmu-miR-9, 429, 200c, 200b, 24	–
*Gsc*	mmu-miR-200c	–	–	–
*Hoxa1*	mmu-miR-23b, 28, 203, 9, 10a, 216a	–	mmu-miR-23b	–
*Hoxa2*	mmu-miR-23b	–	–	–
*Irf6*	mmu-miR-24, 27b, 200c, 200b, 324-3p, 429, 216a,	–	–	–
*Pax8*	mmu-miR-203, 9	–	–	–
*Prrx1*	mmu-miR-203, 205, 375, 9, 10a, 124	–	mmu-miR-3093-3p, 124, 9, 195, 497, 375	–
*Prrx2*	mmu-miR-124, 216a	–		–
*Prkra*	mmu-miR-10a, 10b	–	mmu-miR-10b, 27b-star	–
*Sall1*	mmu-miR-195, 497, 28, 205	–	mmu-miR-195, 497	–
*Six1*	mmu-miR-27b, 200a, 200b, 200c, 205, 375, 429, 10a, 217	–	mmu-miR-217, 7a-2-star, 429, 375, 200a	–
*Six4*	mmu-miR-23b, 24, 27b, 200a, 200b, 429, 497, 195, 9, 10a, 124, 216a, 217	mmu-miR-124, 9	mm-miR-344d, 124, 9, 205-star, 27b, 23b	–
*Tcfap2a*	mmu-miR-195, 200a, 200b, 200c, 375, 429, 497, 137	–	mmu-miR-137, 124-star, 497, 429, 200c, 200b	–
*Wnt5a*	mmu-miR-205, 375	–	mmu-miR-205-star, 200a	–
*Chuk*	mmu-miR-23b, 124, 200a,	–	mmu-miR-124, 23b	–
*Hoxb1*	–	–	mmu-miR-124, 375	–
